# Acanthamoeba keratitis in contact lens wearers in a tertiary center of Tunisia, North Africa

**DOI:** 10.1016/j.amsu.2021.102834

**Published:** 2021-09-13

**Authors:** Ben Abdesslem Nadia, Mahjoub Anis, Seghaier Mohamed Ali, Mahjoub Ahmed, Romdhani Sana, Ghorbel Mohamed, Mahjoub Hechemi, Knani Leila, Krifa Fethi

**Affiliations:** Department of Ophthalmology, Farhat Hached Hospital, Faculty of Medicine of Sousse, Sousse, 4000, Tunisia

**Keywords:** Acanthamoeba keratitis, Contact lenses, Risk behaviors, Corneal scraping

## Abstract

**Purpose:**

To analyze the epidemiological and clinical features of Acanathamoeba keratitis **AK** and to assess the risk factors of this corneal infection in contact lens **CL** wearers in a tertiary center of Tunisia, North Africa.

**Methods:**

We carried out a retrospective study between January 2008 and December 2019 in the ophthtalmology department of a referral center, Sousse, Tunisia. A review of the chart of 248 patients using CL and diagnosed with presumed infectious keratitis was done.

Socio demographic, risk behaviors and microbiological findings in case of AK were analyzed. The mean follow-up was 18 months (1 month–4 years).

**Results:**

AK was diagnosed in 29 cases (11.7% of contact lens wearers with infectious keratitis). These 29 cases were analyzed. The mean age at the time of diagnosis was 33, 44 ± 26, 9 years. Almost of the patients (13; 44.82%) used soft monthly disposable contact lenses. Some risk behaviors related to contact lens wearing were found in our study like not washing and drying hands before CL wear, sleeping in CL, topping off, not respecting the adequate replacement frequency and showering or swimming in CL.

After treatment, visual acuity improved only in 10 cases (34.48%), remained the same in 11 cases (37.93%) and worsened in 8 cases (27.58%).

**Conclusion:**

Clinicians must suspect AK in each CL wearer with suggestive clinical signs to allow earlier treatment and better prognosis. Public prevention messages that encourage CL wearers to respect the hygiene rules should be broadly applicable to each person using any type of CL, to minimize the risk of AK.

## Highlights

• The frequency of Acanthamoaba keratitis has increased worldwide parallely to the widespread of introduction of soft contact lenses.
• Acanthamoeba infection is rare in temperate areas and more common in warm and humid environments.
• The infection can be polymicrobial in 13.79% of cases.
• Many risk factors were identified in contact lens wearers with Acanthamoeba keratitis, including disrespect of hygiene rules.

## 1 Introduction

Acanthamoeba keratitis (AK) is an uncommon disease but a sight-threatning corneal infection caused by Acanthamoeba. The first case was reported in 1974 [1], and though the next decade, the infection was considered very rare. Over the last 20 years, the frequency of the disease has increased dramatically in both the developing and the developed countries [2] and over 3000 cases have been diagnosed in the United State alone [3]. This was noted parallely to the widespread introduction of soft contact lenses (CL). CL are used more and more worldwide in spherical ametropia, astigmatism, keratoconus, orthokeratology and even for aesthetic purposes.

Acanthamoebas are free-living amoebas that are found worldwide in air, dust, and water [4]. A resistance of their cysts to antimicrobial agents including chlorine in tap water and lens desinfection solution is common. That's why lens storage cases can be contaminated in case of contact lens wearing even without corneal infection [5]. Acanthamoeba cysts and trophozoites are able to adhere to all types of contact lenses [6], that are considered as a vector of the disease transmission [7]. Some behaviors of CL wearers increase the probability of developing corneal infection such as poor hygiene, swimming with lenses, water contamination and use of a particular lens solution [8].

The aim of this study was to analyze the epidemiological and clinical features of Acanathamoeba keratitis and to assess the risk factors of this corneal infection in contact lens wearers in a tertiary center of Tunisia, North Africa.

## 2 Methods

We carried out a retrospective study between January 2008 and December 2019 in the ophthtalmology department of a referral center, Sousse, Tunisia. A review of the chart of 248 patients using CL and diagnosed with presumed infectious keratitis was done. Patients were excluded from the study if they were any time known or thought to have had a viral or non-infectious keratitis. In some patients, we made later the diagnosis of herpetic keratitis, which is the main differential diagnosis of Acanthamoeba keratitis. They were then excluded from the study.

Information was collected from medical records including: socio-demographic features (age, gender, profession, and region), type of lenses, type of CL solutions, risk factors or behavior, diagnostic delay, previous treatment, visual acuity at presentation, clinical features of the keratitis, treatment and clinical outcome.

For all the patients, a corneal scrapes was performed without topical anesthetic, utilizing a sterilized spatula, from the leading edge and the bed of the ulcer, and Specimens were directly inoculated onto chocolate agar, blood agar and Sabouraud's dextrose agar.

Corneal scrapings were also put onto slides for gram stain. Plates were incubated at 30 °C and we looked for Acanthamoeba daily. The culture confirmation on non nutriment agar with Escherichia Coli overlay required one to three days. But we should wait for two weeks before concluding to negative culture. The contact lenses, storage cases and lens solution were also analyzed systematically.

After corneal scrapings, we usually treat the patients with fortified eye drops (vancomycine 50 mg/ml 5% + ceftazidime 50 mg/ml 5%) 1 drop/hour. Then the treatment was adjusted according to the results of microbiological results and the response to initial drops.

In cases of confirmed Acanthamoeba kearatitis (clinical and/or microbioligical findings), topical antiamoebic therapy was started. Oral Itraconazole was administrated to some patients. If there is no response to medical treatment and in cases of severe keratitis with spread of the infectious process, penetrating keratoplasty was carried out.

If there was a coinfection of Acanthamoeba and other agents, appropriate anti-microbial treatment was given simultaneously to the patient.

The mean follow-up was 18 months (1 month–4 years).

Statistical analysis was performed using IBM SPSS Statistics software.

**This work has been reported in line with the STROCSS criteria** [9].

**It was submitted with a Research Registery UIN:** researchregistry7059. The hyperlink to the registration is https://www.researchregistry.com/register now#home/registrationdetails/6117e6534ae253001e611ef6/

## 3 Results

From January 2008 to December 2019, there were 248 contact lens wearers with clinical diagnosis of infectious keratitis, who were evaluated. All the patients benefited from a corneal scraping, lens and storage cases were analyzed in 224 cases. One hundred seventy three had positive growth (69,75%) and the remaining 75 (30,24%) negative growth. Gram negative bacilli were found in 101 eyes (40, 72%), Gram positive cocci in 47 eyes (18.95%), fungi in 7 eyes (2,82%) and Acanthamoeba in 29 eyes (11.69%).

Of the 29 cases (29 patients, 29 eyes) of Acanthamoeba keratitis, the corneal scraping was negative in 15 cases and the diagnosis of acanthamoeba keratitis was made thanks to the examination of lenses or their storage case in association with the clinical findings (clinical exam and response to anti acanathamoeba drugs). In two cases, the diagnosis was made on in vivo confocal microscopy which showed Acanthamoeba cysts, appearing as round or oval highly refractile structures, with diameters ranging from 15 to 25 μm. Four cases (13.79%) had polymicrobial infection; the isolated microorganism was pseudomonas aeruginosa in 2 cases, staphylococcus aureus in one case and staphylococcus coagulase negative in other case.

**Fig. 1:**
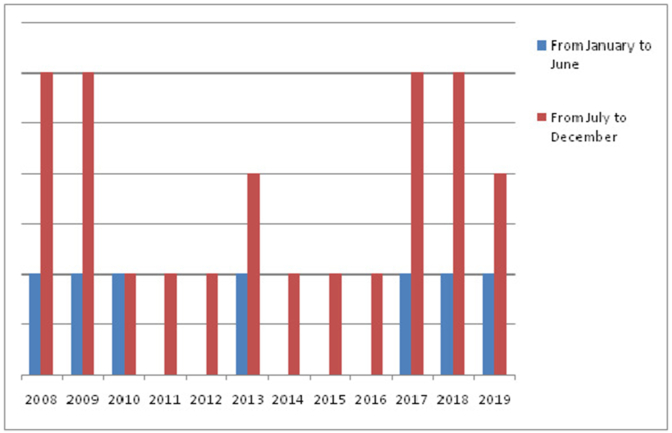
Cases of Acanathamoeba keratitis related to contact lens wear in each year.

The number of cases of AK in each year of our study was shown in Fig. 1. We also counted separately the number of cases from January to June and from July to December in each year of the study (Fig. 1).

Among the 29 patients, 18 were female (62.06%) and 11 were male (37.93%). The mean age at the time of diagnosis was 33, 44 ± 26, 9 years.

A larger proportion of cases (18; 62.06%) were found to have lower primary to upper secondary education and 11 patients (37.93%) had a diploma, university, masters or other professional qualification.

**Sixteen** (55.17%) were from rural region and 13 (44.82%) were from urban region.

**Three** patients (10.34%) were wearing rigid lenses, 7 (24.13%) cosmetic lenses, 4 (13.79%) conventional lenses and 2 (6.89%) soft daily disposable lenses. Almost of the patients (13; 44.82%) used soft monthly disposable contact lenses.

Only 4 patients (13.79%) used hydrogen peroxide solution for disinfecting their contact lenses and 25 (86.20%) used multipurpose solution.

Some risk behaviors related to contact lens wearing were found in our study. Seventeen (58.62%) didn't wash and dry their hands before handling contact lenses, 10 (34.48%) were sleeping overnight in soft contact lenses, 20 (68.96%) were topping off disinfecting solution and 8 of them ever used expired solution, 12 (41.37%) extended the adequate replacement frequency of lenses, 18 (62.06%) were showering or swimming in contact lenses, 7 (24.13%) were rinsing their lenses in tap water and 3 of them were storing CL in tap water. Among the 29 patients, 5 (17.24%) used cosmetic contact lenses of another person.

The socio-demographic characteristics, microbiological findings and risks behaviors related to contact lens wear were resumed in Table 1.

**Table 1:** Socio-demographic characteristics, microbiological findings and risk factors of Acanthamoeba keratitis in contact lens wearers.

	Number of cases among AK	Percentage
Gender		
Male	11	37.93%
Female	18	62.06%
Age		
Less than 15	3	10.34%
15-35	18	62.06%
More than 35	8	27.58%
Education		
Lower primary to upper secondary	18	62.06%
Diplomas, university degrees and other qualification	11	37.93%
Origin		
Rural	16	55.17%
Urban	13	44.82%
Lens type		
Rigid	3	10.34%
Cosmetic	7	24.13%
Conventional	4	13.79%
Daily disposable	2	6.89%
Monthly disposable	13	44.82%
Solution		
Hydrogen peroxide	4	13.79%
Multipurpose	25	86.20%
Risk behavior		
Not washing and drying hands before CL wear	17	58.62%
Sleeping in CL	10	34.48%
Topping off	20	68.96%
Not respecting the adequate replacement frequency	12	41.37%
Showering or swimming in contact lenses	18	62.06%
Rinsing their lenses in tap water	7	24.13%
Using lenses of another person	5	17.24%
Corneal scraping		
Negative	15	51.72%
Positive for Acanthamoeba only	10	34.48%
Acanthamoeba + Pseudomonas aeruginosa	2	6.89%
Acanthamoeba + staphylococcus	2	6.89%

**Fig. 2:**
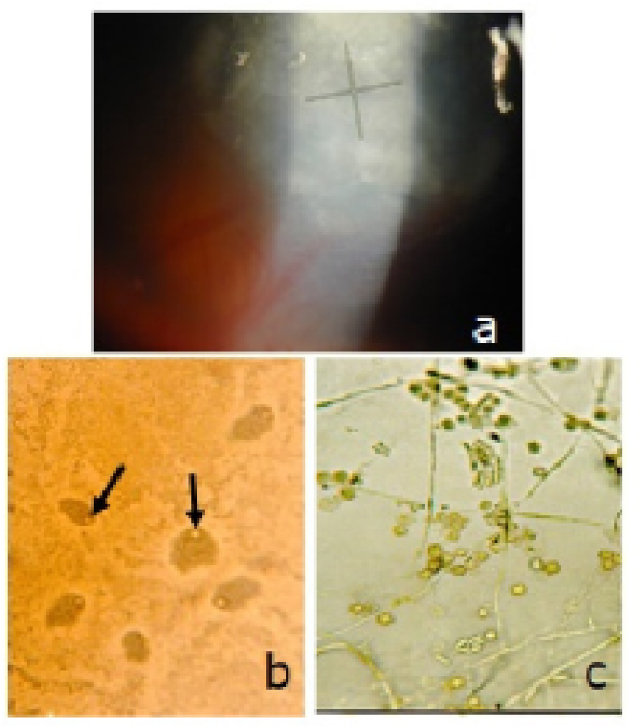
AK in a 24 year-old woman wearing cosmetic lenses. a. Anterior segment photography of the eye showing diffuse stroma infiltrate. b. Conjunctival swab exam: bacteria (Escherichia Coli), trophozoites and cysts of Acanthamoeba Sp. c. Parasitological exam of the lenses and solution of lens storage cases: many cysts of Acanthamoeba Sp.

**Fig. 3:**
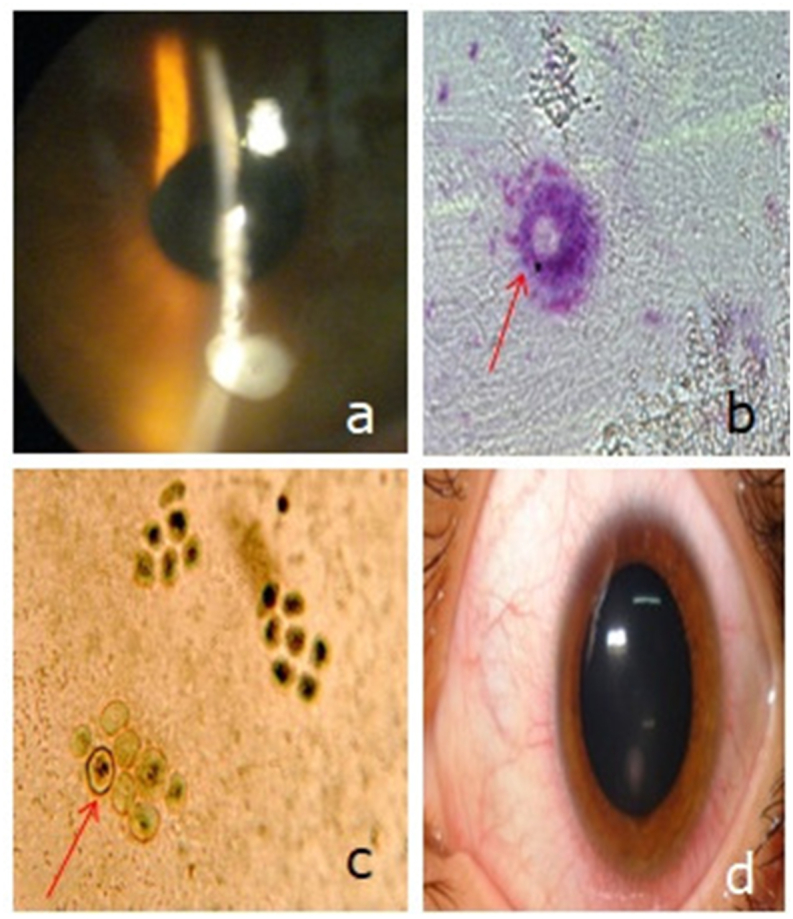
AK in a 33 year-old man with monthly disposable soft CL. a.Anterior segment photography showing a corneal stroma infiltrate. His initial visual acuity was 2/10. b.Parasitological exam of the lenses and solution of storage cases finding the vegetative form of Acanthamoeba c. Parasitological exam of the lenses and solution of storage cases finding Acanthamoeba cystsd. Anterior segment photography of the same patient 6 months after treatment showing an inferior corneal scar. The visual acuity improved to 9/10.

**Fig. 4:**
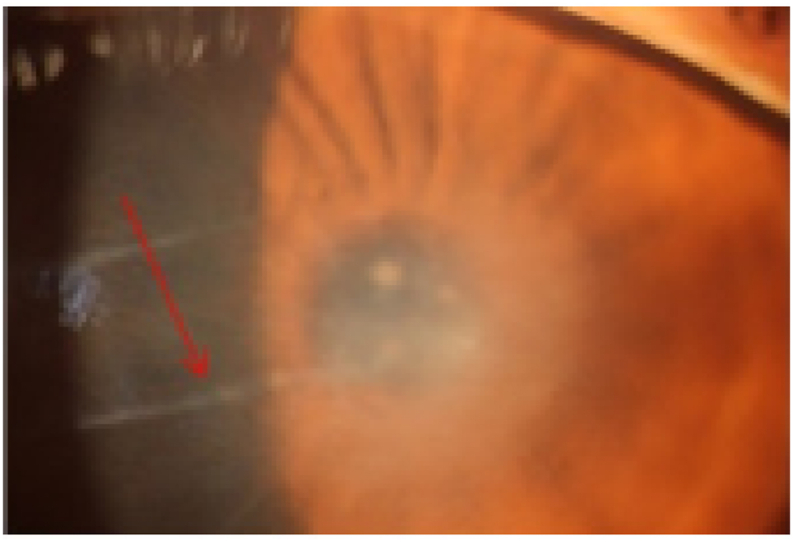
Anterior segement photography of a 29 year-old patient with AK showing keratoneueritis (red arrow). (For interpretation of the references to color in this figure legend, the reader is referred to the Web version of this article.)

**Fig. 5:**
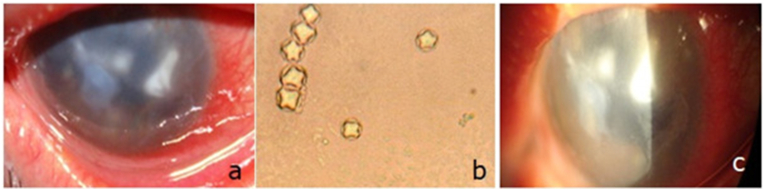
AK in a 44 year-old woman wearing conventional CL for high myopiaa a. Anterior segment photography showing multifocal infiltrateb b. Parasitological exam of the lenses finding Acanthamoeba c. Photography of the anterior segement of the same patient after 2 years, showing a large corneal opacity, the visual acuity was reduced to 1/10. He then benefited from a keratoplasty.

All cases presented to our department with blurry vision, red and painful eye. The slit lamp examination found diffuse stroma infiltrate (Fig. 2, Fig. 3) in 19 cases (65.51%), corneal ulcer in 5 eyes (17.24%), radial keratoneuritis (Fig. 4) in 2 cases (6.89%), hypopion in 7 eyes (24.13%), immunitary ring in 2 cases (6.89%) and multifocal infiltrate (Fig. 5) in 5 cases (17.24%).

Initial visual acuity ranged from light perception to 4/10. It was less than 1/10 in 20 cases (68.96%). An early diagnosis (less than 1 month) was done in 11 cases (37.93%), while a late diagnosis (≥1 month) was done in 18 cases (62.06%). Among the 18 cases of late diagnosis, 7 patients were taking antibiotis, antiherpetic drugs or corticosteroids for a misdiagnosis of bacterial or herpetic corneal infection.

After confirming the diagnosis of AK thanks to microbiological and clinical findings, all the patients received two antiparasitic drops. Because of the unavailability of Polyhexamethylene biguanide (PHMB 0.002%) in our country, only three patients benefited from the association PHMB- Hexamidine as antiparasitic treatment.

Picloxydine dichlorhydrate (Vitabact) or Neomuycin in association with Hexamidine 0.1% (Désomédine) were given for a period ranging from four to six months in 26 cases. Sixteen patients (55.17%) received Fluconazole per os 800 mg the first day then 400 mg/day because of deep stromal infiltrate or hypopion. Four patients underwent a surgey (keratoplasty) because of a corneal scar in 3 cases and corneal perforation in 1 case.

After treatment, visual acuity improved only in 10 cases (34.48%), remained the same in 11 cases (37.93%) and worsened in 8 cases (27.58%).

After the infection, 86.2% of patients with AK (25 patients) discontinued contact lens wear because of fear of having another infection or sight loss.

## 4 Discussion

Contact lenses offer nowadays many optical and cosmetic benefits. There are 140 millions CL wearers worldwide [10]. Over the past decade, an outbreak of serious corneal infection has been recognized amon CL wearers, including Acanthamoeba keratitis [11].

Acanthamoeba keratitis is a serious corneal infection that can lead to visual loss or a need for corneal transplant. In recent decades, AK has been identified as an emerging disease among contact lens wearers.

In our study, an outbreak of AK was found in 2008–2009, because of the start of large use of CL in our country then. There have been published reports in the same period of an increase of AK in multiple countries, especially the United States [12]. After that, the frequency decreased slightly in our study, this can be due to the intervention of our government, ophthalmologists and opticians to make people aware of CL risks and improved behaviour related to CL use. However in recent years, cases among CL wearers have reemerged (since 2017), this trend has also been seen in other developing and develeped countries. The incidence in the United States is 10 time greater than before 2004 [13] and in a recent study in China [14], 50% of cases of microbial kerattis in CL wearers was due to AK. The increasing use of CL worldwide in addition to the improvement in methods of diagnosis of AK and other infectious diseases of the eye made this high incidence [15,16].

In our study, the infection was more frequent in the period of year from July to December each year. This can be due to the fact that wedding parties are usually held in summer (July and August) in our country and people are used to wear cosmetic CL as a guest or as a bride. Moreover, two of our patients were brides wearing colored contact lenses. Another reason of the outbreak of AK in summer and autumn in Tunisia is the climate which is warm and humid in theses seasons. Acanthamoeba infection is rare in temperate areas and more common in warm and humid environments [17] that are favorable for the conservation of Acanthamoeba cysts, while bacterial keratitis typically predominate in temperate climates [18]. The link between contamination rate and environmental distribution of AK has also been described in other studies [19].

Gram negative bacilli like Pseudomonas aeruginosa were the most commonly identified pathogen in our study followed by Gram positive organisms in CL wear, which is in agreement with the results of other studies [20].

It was also reported that coinfection of Acanthamoeba with bacteri was found in 15%–23% of cases [21,22]. In our study, coinfection was seen in 13.79% of cases.

The culture positive rate of Acanthamoeba ranged from 30% to 60% in the literature [23], in our study it was not greater (34,48%). Acanthamoeba usually penetrate the corneal deep stroma and are not found on corneal surface, that's why corneal scrapings often remain negative, especially in late stage if the keratitis or if the patient has been already treated by antibiotics. . The exam of lenses and solution of storage case was crucial as it helped us to make the diagnosis in a large number of cases. Confocal microscopy is a non invasive technique that is useful for the diagnosis in early stage, when the culture is negative. It allowed us to make the diagnosis in 2 cases in our study. Some clinical features are pathognomonic signs of Acanthamoeba keratitis as immunitary ring and perineural infiltrate, but those signs are not always found [24]. Each one was found in only 2 cases of our study.

Despite of the fact that culture remains the gold standard of Acanthamoeba laboratory diagnosis, several PCR techniques are helpful with high sensitivity and specificity [25].

In our study, 7 patients were taking antibiotis, antiherpetic drugs or corticosteroids for a misdiagnosis of bacterial or herpetic corneal infection.

As clinical features can be similar to other infectious keratitis, it is important to consider AK in all cases of CL keratitis [26]. An early diagnosis and treatment is essential to ensure a good prognosis of a such sight-threatening disease. Infortunalely, a substantial delay in the diagnosis of AK was noted in our study and previous reports [27] and the final visual outcome was poor [28]. In another study, 84% of cases have had a diagnosis other than Acanthamoeba keratitis at initial presentation [29].

Anti Acanthamoeba treatment should be started in any contact lens wearer who presents with a corneal ulcer or stromal infiltration while waiting for the microbiological results, in order to avoid the severe complications of this disease.

AK risk factors in CL wearers include non modifiable and modifiable risk factors. The non modifiable are age, gender, socioeconomic level and origin. In our study, AK was more frequent among patients aged between 15 and 35 years, especially females and associated with lower educational level as well as rural origin. However, other studies have shown a link between an increased risk of AK and high socioeconomic level [30]. Rural region is predisposing to Acanthamoeba infection because these areas are supplied with hard water, which improves limescale deposits on pipes and leads to an increased colonisation of Acanthamoeba [31]. Moreover, the use of stagnant waters (citerns..) in rural region can increase the incidence of AK [32].

Other systemic disease can be associated with AK like diabetes [33] and thyroid disease [34]. Poor contact lense hygiene is the most important risk factor of microbial keatitis and especially AK. It has been reported that 66% of complications found in CL wearers were due to poor hygiene practices [35]. Infrequent or omission of lens disinfection is a common risk behaviour [36]. In a study carried out in Madrid, Spain, the practise of not cleaning CL cases presented statistical significant association with Acanthamoeba DNA presence in healthy CL wearers [37]. Not washing hand before handling CL is accounting for 24% of microbial keratitis on a multivariate analysis, it may result in the contamination of lenses and storage cases by pathogen agents [38]. Wearers who are not respecting recommended contact lens replacement schedules are at risk of more complications and eye discomfort [39]. Worn lenses improve Acanthamoeba attachment from biofilm resulting from tear deposits [40]. Topping off solution in the storage cases can increase the microbial growth, and it also decrease the effectiveness of CL desinfection [41]. Showering or swimming with CL and exposure to any source of water when wearing CL are plausible risk factors for Acanthamoeba infection [42]. Tap water is desinfected to prevent gastrointestinal illnesses but it is not sterile and Acanthamoeba is resistent to chlorine, used for desinfection of swimming pool water.

We should avoid swimming with CL, but if one chooses to do it, he should wear a pair of tight-fitting goggles, until desinfecting his lenses at night. Additionally, when we allow the storage cases drying, we decrease the risk of AK [43]. Occasional overnight wear has also been identified as a risk factor of microbial keratitis in our study and in previous reports in the literature [10,44].

Recently, lens supply by internet or mail order has beeb identified as a risk factor of AK, compared with obtaining CL from an optician [30].

Corneal trauma is the most important factor for Acanthamoeba infection. It has been reported that the adherence of Acanthamoeba, to intact epithelium if the cornea could not cause keratitis in animals [45]. Contact lenses can be contaminated with Acanthamoeba cysts or trophozoites and lead to corneal abrasions. Soft contacts lenses, especially silicone hydrogel lenses have a greater risk than hard contact lenses. Daily disposable lenses have a lower risk than extended wear and monthly disposable lenses as they are not kept in storage cases which evict contamination with Acanthamoeba [46].

In vitro studies have found that Acanthamoeba can be resistant to many contact lens solutions [47].

Some multipurpose solution types were associated with an increased risk of Acanthamoeba keratitis compared with hydrogen peroxide or polyquaternium-based solution [48] because of their failure to achieve a suitable level of disinfection in lens storage cases and in the hands of wearers. This was in concordance with the findings of our study. Among multipurpose solutions, complete multipurpose solution is less effective than other solutions [47].

A combination therapy of topiacl biguanides and diamidines is the most commonly treatment used for AK. This regimen decreases the risk of resistance. But as diamidines are still unavailable in some countries like Tunisia, Picloxydine dichlorhydrate (Vitabact) or Neomycin were associated with Hexamidine in our study. Recently, it has found that 0.5–2.5% povidone-iodine solution is more effective against Acanthamoeba in vitro (both trophozoites and cysts) than chlorhexidine [49].

Surgical treatment like keratoplasty has a role for therapy of some complications but not for initial treatment of AK because it is associated with a poor outcome, such as graft rejection and glaucoma.

The visual prognosis remains poor, especially in structures that have difficulties to make an early diagnosis and where hot keratoplasty is rare.

The major limits of our study are his retrospective nature and the fact that the sample might be not representative of all CL wearers. Some details like risk factors and behaviors were potentially missed in our records. Moreover, this study was conducted in one hospital which can result in some bias in the percentage of cases associated with each lens type.

## 5 Conclusion

AK is a challenging infection to manage. It has been an emerging disease in contact lens wearers, observed continuously over the years, with higher incidence in 2008–2009. Clinicians must suspect AK in each CL wearer with suggestive clinical signs to allow earlier treatment and better prognosis. Public prevention messages that encourage CL wearers to respect the hygiene rules should be broadly applicable to each person using any type of CL, to minimize the risk of AK. Campaign strategy including both opticians and ophthalmologists, should be scheduled to raise awareness among CL wearers especially in rural region. Likewise, manufactures of CL ought to innovate lens care solution to reach an effective level of disinfection against Acanthamoeba.

## Fundings

None.

## Availability of data and materials

The datasets used and/or analyzed during the current study are available from the corresponding author on request.

## Ethics approval and consents to participate

Not applicable.

## Sources of funding

No source of funding

## Consent for publication

Not applicable.

## Author contribution

Mahjoub Ahmed, Mahjoub Anis, Seghaier Mohamed Ali and Romdhani Sana collected data, Ben Abdesslem Nadia analyzed data and drafted the manuscript. Mahjoub Hechemi, Leila Knani, Ghorbel Mohamed and Krifa Fethi provided critical manuscript revisions.

All authors read and approved the final manuscript.

## Registration of research studies

Name of the registry:
Acanthamoeba keratitis in contact lens wearers in a tertiary center of Tunisia, North Africa
Unique Identifying number or registration ID: researchregistry7059
Hyperlink to your specific registration (must be publicly accessible and will be checked): https://www.researchregistry.com/register-now#home/registrationdetails/6117e6534ae253001e611ef6/

## Guarantor

Ben Abdesslem Nadia.

## Declaration of competing interest

None.
